# Experiences of Using a Digital Text Messaging Intervention to Support Oral Medication Adherence for People Living With Type 2 Diabetes: Qualitative Process Evaluation

**DOI:** 10.2196/70203

**Published:** 2025-06-06

**Authors:** Nikki Newhouse, Yvonne Kiera Bartlett, Sara Correia Simao, Lisa Miles, Rachel Cholerton, Cassandra Kenning, Louise Locock, Veronika Williams, David P French, Rustam Rea, Andrew Farmer

**Affiliations:** 1 Nuffield Department of Primary Care Health Sciences University of Oxford Oxford United Kingdom; 2 Manchester Centre for Health Psychology School of Health Sciences University of Manchester Manchester United Kingdom; 3 Aberdeen Centre for Evaluation (formerly Health Services Research Unit) University of Aberdeen Aberdeen United Kingdom; 4 School of Nursing Nipissing University Ontario, ON Canada; 5 Oxford Centre for Diabetes, Endocrinology and Metabolism Oxford University Hospitals NHS Foundation Trust Oxford United Kingdom

**Keywords:** type 2 diabetes, SMS, health-related behavior, mobile health, mHealth, mobile phone, qualitative study

## Abstract

**Background:**

Poor adherence to oral medications for type 2 diabetes can increase the risk of health complications. Digital interventions can affect people’s experiences of self-managing a chronic condition, and SMS text messages may provide an effective delivery method for an intervention. The Support Through Mobile Messaging and Digital Health Technology for Diabetes (SuMMiT-D) intervention uses evidence-based SMS text messages to support people with type 2 diabetes with regular and consistent use of diabetes medication.

**Objective:**

This process analysis, conducted alongside a randomized controlled trial of SuMMiT-D, aimed to explore (1) the contextual factors that may interact with the SuMMiT-D intervention and (2) the self-reported mechanisms through which change in behavior or attitude might occur.

**Methods:**

A nested qualitative process study was conducted within primary care in England. A total of 43 trial participants diagnosed with type 2 diabetes were assigned to receive the SuMMiT-D intervention, were undergoing oral glucose-lowering treatment, blood pressure-lowering treatment or lipid-lowering treatment, either alone or in combination, and had access to a mobile phone, took part in semistructured telephone interviews. Data were analyzed using inductive thematic analysis.

**Results:**

In total, 2 overarching themes were developed exploring relevant contextual factors and potential mechanisms of change. The system exerted a range of holistic benefits and supported the cognitions, beliefs, and behaviors necessary for longitudinal self-management. The perceived value of the messages was fluid and linked to contextual need. Appraisal of the system was influenced by existing routines, lifestyle disruption, people’s understanding of type 2 diabetes, relationships with other people, and subjective attitudes toward living with type 2 diabetes in contemporary society.

**Conclusions:**

This work demonstrates the value of engaging people longitudinally in thinking about their general health, the importance of interrogating context, and the holistic benefit of health messaging. Many people perceived wide-ranging and unexpected benefits from using the intervention over time, challenging assumptions about who might be expected to appraise the system more positively and who should be offered access to it.

## Introduction

### Background

Type 2 diabetes (T2D) is a chronic condition characterized by elevated levels of blood sugar, which can lead to long-term health problems such as cardiovascular disease, renal failure, and neuropathy. According to the World Health Organization, the global prevalence of diabetes in 2014 was 8.5% [1]. In the United Kingdom, the number of people living with diabetes reached 5 million for the first time in 2023 [2], with approximately 90% living with a diagnosis of T2D. While lifestyle changes can help control T2D, many people will require medication to lower their glucose levels. Medication adherence is important to help lower the long-term risks of developing complications [3], and even small improvements in glycemic control are associated with a significant reduction in potentially debilitating long-term health complications. However, engagement with self-management is crucial for good glycemic control; people do not always take their medication as intended, and it has been reported that people who are prescribed self-administered medications typically take only approximately half their prescribed doses [4]. Authors of a Cochrane review of interventions aimed at improving medication adherence concluded that they were “mostly complex and not very effective” [4]. In addition, many trials in this area do not use an established theoretical base, which means that they may be missing content with evidence of effectiveness in changing people’s behaviors [5]. As such, there is growing interest in harnessing simple and ubiquitous technologies to deliver evidence-based interventions to address this challenge [6].

A potential low-cost approach to support patients with medication adherence and lifestyle management is the receipt of brief digital messages. Mobile health initiatives have the potential to deliver simple, accessible, scalable, and sustainable support. SMS text messages are often perceived by users as a familiar and user-friendly technology requiring limited digital literacy and operating on any kind of mobile telephone. From a health perspective, SMS text messages have a low per-person cost and are routinely used in primary care services in the United Kingdom. Studies have demonstrated that SMS text messages can remind people about their condition and motivate self-management, and favorable outcomes have long been documented in a variety of contexts, such as short-term weight loss, smoking cessation, hypertension, and chronic illness [7-11]. Furthermore, interventions that use SMS text messages to target behaviors such as medication adherence, diet, and exercise have been found to be effective in lowering blood glucose levels in people with T2D—a systematic review of 111 eligible randomized controlled trials evaluated the effectiveness of telemedicine on glycated hemoglobin in diabetes and found that telemedicine achieved significant reductions in glycated hemoglobin during all 3 follow-up periods (<3 months, 4-12 months, and >12 months) [12].

Nonetheless, SMS text messaging systems used to deliver health information and support are complex digital health interventions, and using SMS text messaging as a vehicle to support self-management requires clear theoretical underpinning and thoughtful message design to be clinically effective and acceptable to users [13] and understand and explain how such a system may exert its effects. Although SMS text messages are generally reported to be acceptable to the user as an intervention format, aspects such as scheduling; tone; and evidence-based, tailored content are important considerations [6]. Similarly, the effectiveness of complex interventions is critically influenced by the context of their implementation [14,15]. Demystifying the assumed complexity associated with contextual variation is vital for understanding the potential strengths and limitations of the intervention in question. Crucially, the chronic nature of T2D and significant health complications associated with poor glucose control point to the need for understanding whether, how, and why self-management interventions work in the long term. To date, there are few studies that explore the interplay between appraisal of receipt of such messages and user context over time [5].

### The Support Through Mobile Messaging and Digital Health Technology for Diabetes Project

The Support Through Mobile Messaging and Digital Health Technology for Diabetes (SuMMiT-D) research program aimed to address these challenges by developing evidence-based messages that are acceptable to the target population and assessing their effectiveness through a randomized controlled trial with 1-year follow-up [16]. To facilitate implementation, the intervention is designed to be delivered to the whole population of people with T2D without involving questionnaires assessing people’s current beliefs and behaviors regarding medication adherence. This required the intervention to include content that would be relevant to people who perhaps intentionally did not take medication as prescribed, as well as those who had occasional unintentional lapses in adherence.

**Figure 1:**
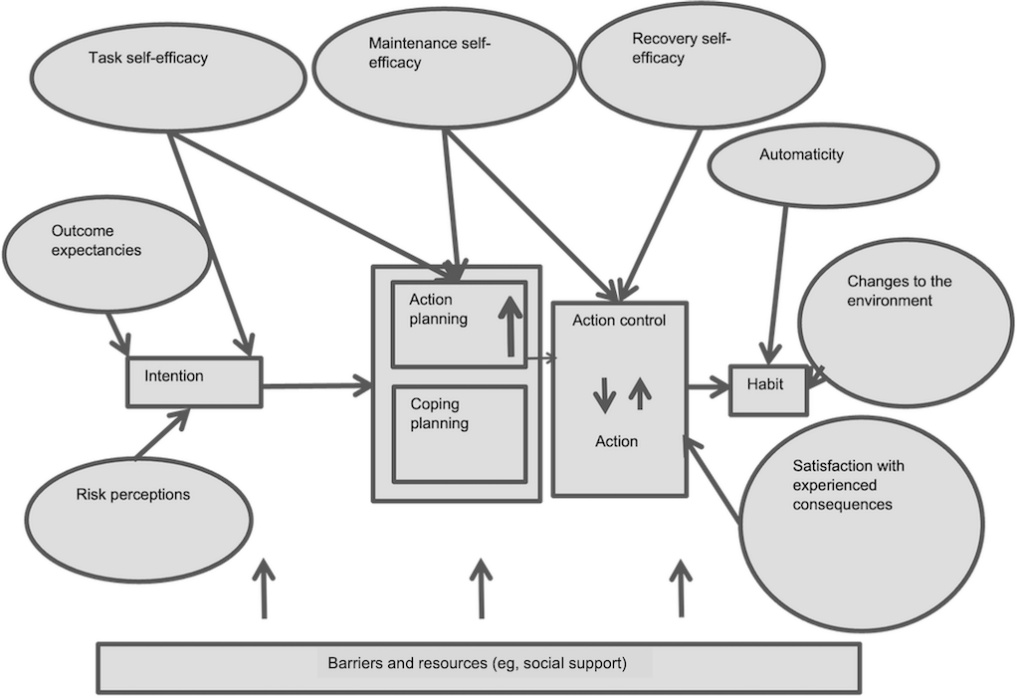
Logic model of the intervention grounded in principles derived from the health action process approach.

Formative work [17] has identified techniques and constructs of interventions that are associated with positive change in medication adherence and mapped these onto a standard taxonomy of behavior change techniques (BCTs) [18]. BCTs have been described as the “active ingredients” of an intervention and include techniques such as problem-solving and action planning. The evidence-based BCTs included were intended to influence cognitions, beliefs, and behaviors associated with medication adherence. They were designed to support intention formation (for those who did not intend to take their medication as prescribed) and then support those who did intend to take medication as prescribed in carrying out this behavior consistently in the long term. SMS text messages were then developed collaboratively with health care professionals, behavior change experts, and people with T2D. Each message was linked to a specific BCT. The messages were assessed for how well they represented their intended BCT and their acceptability to people with T2D. Messages that were not acceptable or did not represent their intended BCT well were removed at each stage of development. A logic model of the intervention was created grounded in principles derived from the health action process approach [19] (Figure 1 [19]) to propose specific constructs that may contribute to how change might occur. This model shows how we designed the intervention to support people in forming an intention to take medication, taking action necessary to take their medication, and monitoring this action to encourage consistency and recover from slips with the aim of supporting habit formation and, thus, medication taking in the long term. The approach taken follows the recommendations of the updated Medical Research Council framework for the development and evaluation of complex interventions to develop such logic models to facilitate process analyses [20,21].

Iterative, mixed methods development work comprising pilot and feasibility studies established that the system architecture was robust and that the intervention and trial processes were acceptable and feasible to participants [22-28]. In addition, formative work confirmed that receiving the messages was associated with increases in key predictors of medication adherence (eg, intention) and that changes in predictors were associated with self-reported medication adherence [29]. The summative trial data were collected from March 2021 to June 2022. Alongside the effectiveness and cost-effectiveness outcomes, data were collected to facilitate a mixed methods process evaluation. This paper describes the qualitative aspect of this process evaluation.

### Qualitative Process Evaluation

Process evaluations aim to understand how complex interventions bring about their outcomes by exploring implementation, contexts, and mechanisms of action [30]. They have three main aims: (1) to understand what is delivered and how, (2) to understand the response to an intervention, and (3) to understand the contextual factors that may influence the effectiveness of an intervention positively or negatively. This analysis is focused on the latter 2 aims and is grounded in qualitative methods, an approach acknowledged as bringing particular value to process evaluations of effectiveness trials [20,31-34]. Using qualitative methods to explore potential mechanisms of action allows researchers to understand why certain mechanisms may work or not work for certain people but also leaves open the possibility of identifying mechanisms of action that had not been previously considered as part of our a priori logic model [35].

### Aim

The aims of this analysis were to gain an understanding of (1) how people’s perceptions of their personal context might be related to their self-management of T2D and how the SuMMiT-D intervention might interact with this and (2) potential mechanisms of action of the intervention by exploring how people reflect upon and describe any changes in feelings and behavior over the course of a year of use of the system.

## Methods

### Design

This qualitative interview study was embedded in the SuMMiT-D summative trial [16]. The qualitative analysis was completed before the quantitative data were analyzed to allow researchers to gain an understanding of participants’ experiences of the intervention and interpret this without being influenced by whether the intervention was clinically effective. This approach also intended to support the exploration of new insights in the subsequent quantitative analysis [33].

### Intervention

The message library contained the BCT-based messages targeting medication adherence as well as information messages related to other aspects of T2D care, including diet and exercise. These information messages were added in response to formative feedback to improve engagement. Table 1 provides examples of intervention SMS text messages and linked BCTs. Participants randomized to the intervention group received up to 4 automated SMS text messages per week related to diabetes self-management and medication taking. The frequency of messages that used a particular group of BCTs could be modified based on a participant’s response to individual messages requesting more or less of that type of message. Messages also encouraged participants to seek further information and included links to external websites such as Diabetes UK where possible.

Potential participants were identified by general practices in England and were provided with a short information leaflet using a variety of methods. Those interested in taking part sent their full name via SMS text message to the trial team to register their interest. Potential participants were also able to call a trial telephone number and receive support in registering. They were then telephoned by a member of the trial team to receive further information about the trial, undergo screening, and confirm eligibility. Participants were randomized to usual care or the intervention after completing baseline measures online. Those randomized to the intervention group received messages for 52 weeks from randomization to the final follow-up. To explore potential intervention efficacy, all participants were asked to complete questionnaires at baseline, 13 weeks, 26 weeks, and the end of their 52-week follow-up period. Medical note reviews were conducted at baseline and 12 months after randomization. The intervention was delivered in 2021 to 2022 during periods of COVID-19–related restrictions; furthermore, the pandemic influenced the delivery of health services as well as people’s routines of daily life nationwide and worldwide during 2020.

**Table 1:** Examples of intervention SMS text messages and linked behavior change techniques (BCTs).

Target and category of message	What the content was based on (eg, BCT, beliefs or concerns, or signposting)	Example message
Medication adherence—BCT	Action planning	“Plan when, where and how you are going to take your medication.”
Medication adherence—BCT	Verbal persuasion about capability	“If you are struggling with your diabetes tablets then don’t worry, you will be able to master it in time. You will get on top of it.”
Medication adherence—BCT	Prompts and cues	“It can be difficult to remember to take your tablets. Why not set an alarm to remind you to take them?”
Medication adherence—BCT	Self-monitoring	“Find a way to split your tablets into days so you notice when you have forgotten to take your tablets.”
Medication adherence—BCT	Social support—practical	“How often do you forget to take your tablets? Take control. Ask your friends and family members to help. Their reminders could help you to improve your diabetes.”
Medication adherence—BCT	Social support—emotional	“If you’re not taking your tablets as often as you should, try discussing your feelings with someone.”
Medication adherence—BCT	Mental rehearsal of successful performance	“Visualise in detail how you will take your tablets tomorrow. This will make it easier when you actually take them.”
Medication adherence—beliefs and concerns	Health care system–related concerns	“Lots of questions? Check who the best person to see might be.”
Diet management	Signposting	“Stuck for new ideas? You can search recipes for mains, desserts and snacks online at Diabetes.org.uk.”

### Participants

Participants were eligible to take part in the SuMMiT-D trial if they were undergoing oral glucose-lowering treatment, blood pressure–lowering treatment, or lipid-lowering treatment either alone or in combination; had access to a mobile phone with which they could send, understand, and retrieve brief SMS text messages in the English language (independently or with support); and were aged ≥35 years—the intervention focused on a broad range of individuals with T2D, but those with earlier-onset diabetes and using insulin alone were not included as these features often require different care pathways (the full inclusion and exclusion criteria are listed in the trial protocol [16]).

Two subgroups of participants allocated to receive the intervention were purposively sampled to gain a richer understanding of the contexts and mechanisms that might contribute to the longitudinal use and acceptability of the SuMMiT-D system: group 1 (n=23), who were interviewed 4 weeks after randomization and again following completion of follow-up measures, and group 2 (n=20), who were only interviewed following completion of follow-up measures, selected to explore emerging themes from preliminary analysis of subgroup 1’s postrandomization interview data. A total of 34 people took part in entry interviews, and 23 (68%) were able to participate in follow-up interviews. Only paired data (ie, entry and exit interview data from 1 person) from group 1 were included to probe use over time through reference to entry interview data.

### Ethical Considerations

This qualitative interview study was approved by West of Scotland Research Ethics Committee 5 (20/WS/0103). Participation in the qualitative study was optional, and informed consent was obtained at the point of recruitment to the main study. All qualitative data were appropriately deidentified. Participants were not reimbursed or paid for taking part.

### Recruitment and Interview Procedure

Participants were invited to take part using information provided at the point of recruitment to the main study, and no participants were previously known to the interviewers. A purposive approach was adopted to ensure a varied sample across specific factors identified in formative work as having the potential to influence engagement and acceptability, including but not limited to (1) ethnicity, (2) equal gender split, (3) age ≤50 years, (4) diagnosis of <1 or >10 years, (5) a medication change in the previous 3 months, (6) use of a standard mobile phone or no description of any other technological devices in baseline questionnaires, (7) participation in a T2D education program, (8) Medication Adherence Report Scale [36] score of <20, and (9) living in an area with a deprivation score of >30.5.

Semistructured interview schedules were codeveloped by experts in health psychology (YKB, DPF, LM, and RC), human-computer interaction (NN), and qualitative methods (VW, LL, NN, and YKB). Pragmatic content analysis of formative qualitative data identified potential contextual factors and mechanisms hypothesized to influence how the SuMMiT-D intervention works on an individual level. Subsequently, questions were developed to elicit data about the experience of living with T2D, the experience of using the SuMMiT-D system over time, and the contexts and perceived mechanisms of use described by participants. In accordance with guidelines related to the potential for measurement itself to affect trial outcomes within trials [37], we took steps to mitigate the potential impact on outcome data of participating in these interviews. Entry interviews took place 4 weeks after randomization to allow for exploration of initial changes in response to the messages while still leaving an appropriate gap between baseline measurement and the interview. Similarly, all interviews at 52 weeks were conducted following quantitative data collection. The interviews were conducted via telephone to minimize participant burden (and in accordance with any COVID-19 social distancing guidance in place at the time of the interview), and no other people were present. The group 1 interviews took place between April 2021 and August 2022, and the group 2 interviews took place between July 2022 and September 2022. Interviews were conducted by NN, YKB, LM, and RC, all of whom are experienced nonclinical researchers. Each interview was audio recorded, and reflective field notes were completed afterward to record any thoughts. Audio recordings were transcribed verbatim, and the transcripts were checked and anonymized.

### Positionality of the Research Team

The research team brought together a diverse range of expertise and experiences, including trial design, complex intervention evaluation, clinical practice (nursing, respiratory health, and T2D), health service research, health psychology, and human-computer interaction. Team members had extensive experience in qualitative methodologies, enhancing the rigor and reflexivity of the analysis. The team comprised individuals at varying career stages and of varying ages working across different regions of the United Kingdom, including urban, rural, affluent, and socioeconomically deprived areas. This diversity contributed a broad range of perspectives to data interpretation. Interviews were conducted by a subset of the team, all experienced qualitative researchers who reflected on their own positionality throughout data collection and analysis. The varied disciplinary backgrounds and personal characteristics of the team helped surface multiple interpretations, enriching the depth and credibility of the findings while recognizing the potential influence of our positions on the research process.

### Patient and Public Involvement

Patient and public involvement (PPI) representatives have been involved throughout the SuMMiT-D program of work. A panel of 11 members with experience of living with T2D or caring for someone living with the condition was set up at the formative stage of the program and continues to inform our work—all participant-facing paper and online documents for the SuMMiT-D trial were reviewed by PPI panel members, and main findings were discussed with the panel to facilitate our interpretation of the data. Recruitment progress and key information were made available to the PPI group and wider public in the form of a newsletter and as part of a dedicated website.

### Analysis

A thematic analysis approach was taken [38], chosen due to its theoretical and epistemological flexibility and recognition that the researcher plays an active, interpretive role in identifying and reporting themes [39]. All transcripts were uploaded to qualitative data management software (NVivo; version 12; QSR International) and analyzed thematically taking an experiential orientation, which prioritizes the examination of how a given social reality may be experienced by the participant [40]. Two researchers (NN and SS) initially independently coded 6 transcripts and then compared and integrated their codes. Building on a pragmatic content analysis of formative qualitative data collected throughout the wider SuMMiT-D project [41] and team discussions, central organizing concepts and themes were identified, resulting in a hierarchical coding framework. The coding framework was used to code the remaining transcripts, and codes were added or subsumed to reflect the developing analysis. All coding was reviewed by YKB. An audit trail in the form of detailed field notes and collaborative researcher triangulation supported the rigor of the analysis.

## Results

### Participant Characteristics

In group 1, 68% (23/34) of the invited participants took part in a follow-up interview. In group 2, 54% (20/37) of the invited participants agreed to take part in an interview. Table 2 shows the sociodemographic and group details of the sample.

**Table 2:** Sociodemographic characteristics of the aggregated sample.

	Baseline (n=34), n (%)	Exit sample (n=43)^a^, n (%)
Age ≤50 years	8 (24)	9 (21)
Female	17 (50)	23 (53)
White British	22 (65)	29 (67)
Recently diagnosed	6 (18)^b^	9 (21)^c^
Time since diagnosis >10 years	11 (32)	5 (12)^d^
Use of a standard mobile phone	3 (9)	8 (19)
MARS^e^ score <20	12 (35)	11 (26)
Living in an area with a deprivation score >30.5	8 (24)	10 (23)
Medication changes in the previous 3 months	5 (15)	3 (7)^d^

^a^In total, 68% (23/34) of the participants of the initial sample completed the exit interview. An additional 20 participants completed an exit interview.

^b^Defined as a diagnosis of <1 year.

^c^Defined as a diagnosis in 2019 for the additional sample.

^d^Criteria were only reported for the paired sample (ie, the sample that had entry and exit interview data).

^e^MARS: Medication Adherence Report Scale.

### Themes

#### Overview

In total, 2 overarching themes were identified: first, “What my life looks like,” which explores a range of relevant contextual factors and, second, “Changes in self-management of T2D over time: what and how,” which illustrates perceived changes and potential mechanisms of change as described by the participants. Participants in the SuMMiT-D study reported direct changes in their behavior but also changes in thoughts and feelings regarding their diabetes and self-management as a result of receiving the messages. The analysis broadly challenged a priori assumptions about who might be “expected” to find the system more useful. For example, it was hypothesized based on our formative work that a system specifically targeting medication adherence behaviors may be valued more by people who had been recently diagnosed with T2D or those who had limited social support. However, the long-term passive receipt nature of the system in combination with the complex interaction of self-management of T2D and personal context led to value being perceived across the sample. Similarly, participants who did not perceive value in the system could not be easily grouped by anticipated factors (such as time since diagnosis). Where illustrative quotes are presented, participant gender, age, and time since diagnosis are included to help understand the quotes in context. Themes, subthemes, and illustrative quotations are provided in Table 3.

**Table 3:** Thematic map with themes, subthemes, and illustrative quotations.

Theme and subtheme		Example quotation
“**What my life looks like”**		
	Existing routines and the impact of disruption	“I am hopeless. If a routine changes for me, that’s it, I’m like off the rails.” [Female participant; aged 56 y; >5 y since diagnosis]
	People’s understanding of their condition	“More worried about taking my Metformin than probably any of the other drugs that I’m on.” [Male participant; aged 61 y; >10 y since diagnosis]
	Relationships with others	“I mean they seem to concentrate more on the insulin dependent ones and us tablet controlled ones seem to be left to our own devices.” [Male participant; aged 62 y; >5 y since diagnosis] “I mean if we’re out and something is available to eat, she gives me the nod if I should eat it.” [Male participant; aged 80 y; <5 y since diagnosis]
	What it “means” to live with T2D^a^	“I’ve got it, can’t do anything about it, it’s under control, I’m not worried about that.” [Female participant; aged 73 y; >10 y since diagnosis] “I always call it the fat diabetes when I say I’ve got diabetes, it’s not insulin, it’s the fat one.” [Female; aged 56 y; >5 y since diagnosis]
**Changes in self-management of T2D over time—what and how**		
	The “drip drip” effect	“I do actually think it was an incredibly productive way of engaging with people, because it was ‘light touch.’” [Male participant; aged 50 y; >10 y since diagnosis]
	Take what you need—and stick with it	“No, no nothing was unhelpful. I enjoyed getting them and where it was needed, I changed things. But there wasn’t a lot to change because I was doing the right thing and I realised that because of the messages.” [Female participant; aged 74 y; >5 y since diagnosis]
	The system as a vehicle between then and now	“The variety of messages was good the way they suggested different ways to remind yourself. Make a note of it somewhere, ask someone, make sure a family member is watching over you if you don’t think you’re going to remember. I think there was about nine or 10 different ways, but you can only say it so many times. By differently wording it sometimes—as I said before—it makes you read it again.” [Male participant; aged 54 y; >5 y since diagnosis]

^a^T2D: type 2 diabetes.

#### What My Life Looks Like

##### Overview

Participants’ preexisting thoughts, feelings, and actions regarding T2D, medication adherence, and wider self-management could all be expected to influence how the intervention may have been experienced. Participants described broad and highly subjective contextual factors that influenced their medication adherence and T2D self-management before the intervention, including existing routines and the impact of disruption, their understanding of their condition, relationships with other people, and what it means to live with T2D in contemporary society. While acknowledging that these contextual factors and the understanding of them is fluid, we identified 4 key subthemes that particularly informed our understanding of how the intervention was received by participants.

##### Existing Routines and the Impact of Disruption

Most people described being confident in the self-management of their oral medications, having established routines in place before participating in the SuMMiT-D study, regardless of factors such as time since diagnosis or medication volume. People supported their medication routines using tools such as dosette boxes, mobile apps, and reminders or strategic placement of medications in the home:

Tablets wise, I am very good on doing the tablets. It’s first thing I do in the morning. I get the tablets out, I take them in the morning, I take them in the evening. I’ve got two little jars.

Female participant; aged 73 y; >10 y since diagnosis

Although participants described minor lapses in medication adherence, this was not perceived as problematic, and indeed, adherence was frequently described as easy compared to other self-management and lifestyle factors. Some participants did not know what particular medications were for or believed that their medications were not diabetes specific, and some described not taking them as prescribed. Broadly, medication was perceived as “manageable” and a treatment responsibility that could be “outsourced” to health care professionals. As such, the system’s primary focus on medication adherence was perceived as frustrating or limited by some people but as a helpful reminder or reassurance of “good behavior” by others.

Although participants described medication taking as the cornerstone of T2D self-management, adherence routines were highly susceptible to disruption. Participants described the negative impact of professional workloads, caring responsibilities, bereavement, moving house, religious fasting, shift working, illness, seasonal shifts, low mood, weekends and holidays, COVID-19, changes to medication regimens, irregular eating patterns, and even spontaneity on medication adherence and general self-management:

I am hopeless. If a routine changes for me, that’s it, I’m like off the rails.

Female participant; aged 56 y; >5 y since diagnosis

A lack of symptoms could also throw a routine off course, and while some people had established ways of making sure that they were not caught out (such as keeping extra medication in the car), many described managing mini “cycles” of lapses in which medication was taken late or “topped up” at the next dose. For people who experienced such lapses, the messages proved to be useful in getting medication adherence routine back on track quicker.

##### People’s Understanding of Their Condition

Participants entered the study with differing levels of understanding of and confidence in their ability to manage their diabetes. Some participants described a thorough knowledge of physiological specifics, whereas others seemed confused about the trajectory that the condition might take. Some participants had attended structured education related to their diabetes, but people of working age or those who were diagnosed around the time of the pandemic found access to such courses difficult or absent. People talked about accessing information and developing knowledge about T2D in a variety of ways, and although many described proactive information seeking, a key narrative thread emerged regarding the perceived extent of missing or contradictory information leading to unmet information needs:

Finding good information seems to be difficult. It’s useful to have a link that will point you in the right direction. You put in a Google search, you come across so many links, and then you sit there looking through each and every one of them.

Male participant; aged 62 y; >5 y since diagnosis

Unmet information needs and gaps in knowledge led to people being disproportionately fearful of negative outcomes that might result from unintentionally doing the “wrong thing.” One participant described how he was “more worried about taking my Metformin than probably any of the other drugs that I’m on” (male participant; aged 61 y; >10 y since diagnosis). This led to a general willingness to engage with T2D resources, particularly those that came from trusted sources and provided novel information in an accessible format. The SuMMiT-D system included messages targeting lifestyle factors such as diet and exercise as well as medication adherence, and these “little nuggets of information” (female participant; aged 44 y; <5 y since diagnosis) were appraised positively by nearly all participants.

##### Relationships With Others

People described their perceptions of professional and personal support and how this influenced the way in which certain messages were received. The desire for information described previously was closely linked to perceived limitations in the amount and quality of care received from health care professionals or an unwillingness to place further burden on an already overstretched health system. Participants were recruited through their general practitioner throughout England from areas within all Index of Multiple Deprivation deciles, and descriptions of “usual care” differed widely. Some people described positive experiences with health care teams, but others described an absence of general practitioner contact, which was particularly surprising to the newly diagnosed and was also felt to be illustrative of the dissonance between the perceived severity of the condition and the treatment provided:

I mean they seem to concentrate more on the insulin dependent ones and us tablet controlled ones seem to be left to our own devices.

Male participant; aged 62 y; >5 y since diagnosis

This was compounded by a broad expectation that people should be responsible for self-management that required specialist knowledge or resources, such as purchasing and using blood pressure monitors (but, conversely, that people should not be encouraged to check blood sugar levels). In addition, care was often described as perfunctory and adhering to a fixed protocol. For example, one participant described a diabetes nurse repeatedly wanting to prescribe statins “because you’re diabetic” despite other health care professionals insisting that this was not necessary.

Participants generally described a fundamental lack of engagement with their health care teams and felt that they were treading water rather than being supported to make meaningful change. This lack of relationship led to brief, uncomfortable interactions grounded in what was perceived to be a paternalistic view of T2D as a lifestyle problem in which participants described feeling blamed for their condition:

I don’t really like my diabetes nurse or any of them...because they’re incredibly patronising. Oh, they treat you like you are six years old and a little bit stupid as well...And the way they speak to you it’s just like, “Oh, why are you being so bloody lazy,” yeh. They won’t say it in so many words, but you do understand what the connotations is, yeh.

Female participant; aged 53 y; >10 y since diagnosis

People’s personal relationships were crucial in supporting their self-management, although again, experiences and preferences regarding the type and degree of social support differed. For example, many people relied on others for support regarding food planning and preparation, with wives, daughters, and mothers frequently mentioned as taking responsibility for ensuring that the household diet was diabetes compliant:

I mean if we’re out and something is available to eat, she gives me the nod if I should eat it.

Male participant; aged 80 y; <5 y since diagnosis

People discussed learning about diabetes management from friends and family who were also living with T2D or who had medical training:

My parents were diabetic, and my father actually went blind through the diabetic neuropathy and macular degeneration...I’ve seen that. So obviously I am not going to jeopardise my eyesight...So, I’m particular about it.

Female participant; aged 55 y; >10 y since diagnosis

As a result, the diversity of personal circumstances led to a range of responses to SMS text messages that invited consideration of others’ feelings (about one’s potential deterioration caused by poor T2D self-management) or ways in which other people might be involved in supporting self-management. For some participants who lived alone, these kinds of messages served to highlight their isolation; some participants had negative reactions to messages suggesting they ask for support from friends and family, with these messages perceived as “nannying.” However, others described how the “friends and family” messages encouraged them to talk to others about their T2D, sometimes for the first time; others described how the messages had encouraged the understanding of the importance of taking responsibility for their own health and well-being:

Well, you know it helps concentrate your mind on you know, with the text messages you realise that it’s up to you to sort it out. It’s nobody else. It’s entirely your life, and you’ve gotta sort it out for yourself or whatever. And that encouraged me to sort of make the changes that was necessary.

Female participant; aged 68 y; <5 y since diagnosis

##### What It “Means” to Live With T2D

Participants described how managing a chronic condition had challenged their own personal narratives about what it “means” to live a healthy life. These narratives were often informed by a perceived trade-off between the effort required to maintain glycemic control and navigating real life. The everyday impact of diabetes differed broadly:

I think it’s a nuisance now. Yes, it controls your life.

Female participant; aged 55 y; >10 y since diagnosis

I’ve got it, can’t do anything about it, it’s under control, I’m not worried about that.

Female participant; aged 73 y; >10 y since diagnosis

However, the labor of self-management was widely acknowledged—the need for consistent experimentation with and self-monitoring of diet, the boredom of always thinking about T2D, the times when lapses in self-management (often unintentional) had led to frightening episodes of uncontrolled blood sugar, and the difficulty of managing what could be an invisible condition:

And because diabetes...it doesn’t hurt you today, yeh; not at all. So, I find...this is the reason why I’ve been struggling for years to acknowledge it really; I didn’t do anything about it. Because what does it matter, yeh, it doesn’t make me feel ill.

Female participant; aged 53 y; >10 y since diagnosis

The effort of self-management for many participants was compounded by an awareness of negative societal narratives regarding T2D. Some people talked about the shock of being diagnosed with a condition heavily linked to assumptions regarding the “kind” of person that is diagnosed. For example, some described struggling with “the fact that somebody’s just told you that you’re a fat bastard and you need to do something about it” (female participant; aged 68 y; <5 y since diagnosis) or said that “I always call it the fat diabetes when I say I’ve got diabetes, it’s not insulin, it’s the fat one” (female participant; aged 56 y; >5 y since diagnosis). One participant who had been diagnosed at the age of 40 years described how an acquaintance commented that she “didn’t fit that profile” (female participant; aged 40 y; <1 y since diagnosis). Rather than being keen to engage with self-management tools or information, people described how this need to realign one’s notion of the self with negative social stereotypes could lead to initial medication refusal, nondisclosure of the condition to others, and feelings of shame:

I’ve never been ill before so that was a big thing with me in the beginning...prior to being told I’m a diabetic I was like a fit 40 years old, cycling 60 miles a day, walking at least 25 miles a week, things like that. I’m looking after a big garden, I’m also a carer. I did all that, a piece of cake, and all of a sudden, I’d gone down to a 90-year-old in a lot of pain.

Male participant; aged 69 y; <5 y since diagnosis

What followed the initial diagnosis was highly subjective. Increasingly, a narrative thread emerged regarding what “kind” of person people perceived themselves to be in the context of self-management. For example, some people talked about being highly motivated and disciplined “in general,” whereas others perceived themselves as “lazy” or “chaotic.” Furthermore, participants talked about the fluidity and evolution of their self-management over time, especially if they had the capacity to experiment with food and routines. Improved self-awareness and increased knowledge sometimes led to changing definitions of success, and while the notion of “good” self-management was associated with autonomy and the confidence to understand subjective norms, this did not necessarily equate to compliance:

To be still able to carry on with my life as I want it to be. That is, you know. I’m under no illusion, I will never run the London marathon or anything like that, not even before I was, you know that was completely out of the question. I just want to be able to live life as I like it.

Female participant; aged 53 y; >10 y since diagnosis

Differences in self-perception impacted how self-management was “operationalized” and, therefore, how the SuMMiT-D SMS text messages were interpreted. Some people liked the idea of just being told what to do, whereas others found that patronizing or insufficient.

#### Changes in Self-Management of T2D Over Time: What and How

##### Overview

Participants reported a variety of changes in their behavior, emotions, and cognitions that they attributed to receiving the SMS text messages. These included improvements in consistency in taking oral medications in general, changes made to their diet (eg, reducing sugar intake or reducing portion sizes), increases in physical activity, and talking to people about their diabetes more. One participant reported stopping smoking during the intervention, saying that the messages had prompted an increased general focus on his health. Other participants described purchasing and using smartwatches for self-tracking or taking up specific forms of exercise. People described changes in emotions and cognitions about living with T2D, for example, feeling more proactive about self-management, feeling reassured that existing behaviors were being reinforced, or experiencing a change in their overall recognition of responsibility for their health. The intervention was designed to operate along multiple potential mechanisms of action to try to ensure that there was something included for people with a range of beliefs, behaviors, and experiences regarding their medication adherence and general T2D self-management. These mechanisms were broadly classified as shifts in behavior, cognitions, and feelings. Crucially, patterns in the way in which people talked about these mechanisms in the entry interviews were described in similar ways in their exit interviews, indicating that initial perceptions and experiences of change may have been maintained over the course of the year of interacting with the system. In total, 3 subthemes were identified that propose potential mechanisms of change brought about by both the intervention content and format.

##### The “Drip Drip” Effect

Many participants talked about the unobtrusive way in which the SMS text messages prompted them to remain engaged with their T2D self-management. The consistent, passive receipt nature of the system over time was acknowledged as repetitive and simple:

I do actually think it was an incredibly productive way of engaging with people, because it was “light touch.”

Male participant; aged 50 y; >10 y since diagnosis

Some participants did report following specific advice contained within the messages (eg, leaving medication in the car or setting an alarm on their phone to remind them to take medication following messages that specifically suggested these actions), which could indicate that this information was either novel or “landed” with them in a novel way, so they felt able to follow it. Some participants directly reported feeling more knowledgeable about their diabetes and having learned things from the messages (or linked websites). However, many participants described the messages as containing limited novel information but that it was their *consistent presence* that was perceived to be helpful. Rather than simply or routinely following specific advice within the messages, participants described how receiving the messages resulted in (1) an increase in the personal salience of diabetes, (2) improved awareness of when they were or were not managing their diabetes well, and (3) reinforcement of the positive steps they were taking. People described changes that they perceived as significant, such as taking medications consistently when on holiday instead of having some time off or noting how their medication taking in particular had become more consistent over the course of the year:

...some of them were a lot of ones about remembering to take your tablets and not missing your doses and things like that, which were good, they didn’t annoy me at all but they were kind of the drip-drip ones, which I think when you reflect on it probably did make you think more about taking your tablets, and making sure you took your tablets.

Male participant; aged 43 y; >5 y since diagnosis

Many participants also described how receiving the messages over time made them feel cared for and supported. This was especially relevant in cases in which positive relationships with health care teams were difficult, support was absent, or living conditions were challenging. One participant referred to the system as being “like having a friend text you on the phone basically—sort of like sending you encouragement...keeping me on my toes” (male participant; aged 55 y; >5 y since diagnosis). The sense of being looked after and reassured that T2D self-management was within their control had the effect of encouraging people to accept the reality of self-management and also served to reduce the stigma felt by some people about “admitting” to living with T2D:

It makes me realise that I need to be responsible for my health. It made me feel that people care if they go to that extent of texting me every day. It makes me feel cared for and feel important.

Female participant; aged 59 y; <5 y since diagnosis

This potential mechanism of action shows the strength of the combination of content and delivery in this intervention. Specific BCTs were included to encourage direct change in action (eg, prompts and cues) or support people in maintaining changes (eg, self-monitoring and problem-solving), and these appeared to work in combination with the consistent, light-touch delivery to support people in changing. Furthermore, participants described how the messages encouraged them to acknowledge the potential negative consequences of poor self-care and reported improved understanding of the risks involved in not managing their condition. For some people, discussion about the condition with others was an important part of this consolidation of understanding, and many participants talked about saving messages and returning to them to support discussion with others, even forwarding the messages to people they knew whom they thought might benefit from the information. Overall, these effects enabled participants to either maintain positive behaviors in situations in which these would previously have been disrupted or make changes to their behaviors to better support their self-management:

Yeah, my intentions are much more stronger or even there more than they even would have been, if you see what I mean? I wouldn’t have cared, I would have just carried on as life, but it is in the back of my mind all the time.

Female participant; aged 56 y; >5 y since diagnosis

##### Take What You Need and Stick With It

For the participants who perceived a benefit to using the system, there was acknowledgment that not all the messages were immediately relevant but that they were happy to receive them and act on the ones that they found useful. One participant compared this to reading a newspaper, where they would simply turn the page past anything that was not of interest. For those participants who did not perceive any particular benefit to using the system, it appeared that this threshold of intermittent reinforcement was simply not met:

I honestly didn’t really find them very helpful. The majority seemed based around sort of getting people to help you and how you can take your tablets and things which I mean, I don’t have any problem with. It seemed to be at people that I don’t know, that didn’t realise they had the condition, or they were you know, not very good at taking tablets or the elderly, something like that.

Female participant; aged 64 y; <5 y since diagnosis

As a result, some participants elected to stop receiving the messages, and others explicitly stated that they would have stopped had they not been part of a research study. Although formal exit interviews were not conducted with participants who withdrew early from the intervention arm of the study, brief feedback gathered by the trial team indicated that the primary reason provided to explain early withdrawal centered on a perceived lack of relevance or usefulness. However, there was no evidence that contextual factors such as length of time since diagnosis, gender, or age determined or predicted whether people would perceive benefit from the system or, conversely, choose to stop receiving the messages. People described the messages as simply “landing” differently depending on their mindset at the time. Furthermore, it became apparent that most of those participants who elected to keep receiving messages for the full year *even though* not all of them were immediately useful or relevant found them to be of benefit on balance. Although some people talked about becoming “immune” to the messages over time as their novelty or impact wore off, others acknowledged that the messages had not seemed to be immediately useful but that they became more valued over time as a “prodder reminder” (male participant; aged 50 y; >10 y since diagnosis), with one participant saying that “I actually miss them really” (male participant; aged 55 y; >5 y since diagnosis). The perceived value of the messages was intimately linked to whatever was subjectively valued by the participant *at that time*. Therefore, this perceived value was a fluid concept—for example, people sometimes needed specific information that was provided via a link to an external website, other people lapsed briefly with their medication and described how the messages reminded them to get back on track, and other people valued the reassurance that came from messages that reinforced their existing behaviors:

No, no nothing was unhelpful. I enjoyed getting them and where it was needed, I changed things. But there wasn’t a lot to change because I was doing the right thing and I realised that because of the messages.

Female participant; aged 74 y; >5 y since diagnosis

##### The System as a Vehicle Between Then and Now

Formative work identified factors of the system and message format that were hypothesized to be important contributors to the intervention’s overall acceptability. These included message novelty, relevance, variety, usefulness, tone, and specificity. Trialing these same factors over an extended period of a year yielded important further insights, and additional factors of regularity and repetition were added to this formative knowledge. The perceived value of the messages was a fluid concept and was linked to contextual need, and the same can be said for system factors. For example, although some participants talked about how the messages became mundane and less relevant over time as foundational habits were formed, the same people also described how the usefulness of these messages shifted and evolved and they were now seen as highly reinforcing of existing behaviors (which had not necessarily been in place at the start of the study). Previous, shorter formative work simply did not examine this shift over time and, thus, perhaps overemphasized the importance of novelty and relevance as a primary mechanism of change. Variety of message content was valued, and furthermore, people talked about the value they attributed to reading the same core message presented in different ways:

The variety of messages was good the way they suggested different ways to remind yourself. Make a note of it somewhere, ask someone, make sure a family member is watching over you if you don’t think you’re going to remember. I think there was about nine or 10 different ways, but you can only say it so many times. By differently wording it sometimes—as I said before—it makes you read it again.

Male participant; aged 54 y; >5 y since diagnosis

The factor of usefulness was a highly subjective concept—the system appeared to act largely as a reminder of the pros and cons of maintaining consistent self-management, and as described previously, people took what they needed from the system and formed their own flexible composite of “useful.” This often included a perception of the messages as positive in tone and specific in terms of information (eg, links to advice on how to read food labels) if those specific factors were important in the moment. Information specificity versus perceived message “abstraction” was a particularly important factor in maintaining engagement; for example, some messages were framed as questions that some participants found confusing. This was compounded by participants perceiving that they needed to respond to these questions and then receiving an automated error message. Furthermore, messages that invited participants to think about hypothetical contexts were consistently described as being similarly abstract and distracting:

Hi, [name], compare yourself to other people who are taking diabetes tablets. Are you more or less likely than them to take your tablets as prescribed? So, that’s a bit subjective really because how would I know if I would be more or less likely to take them...because I don’t know who they are.

Female participant; aged 77 y; <1 y since diagnosis

The factors of regularity and repetition were important system factors described as contributing to engagement and consolidation of information and understanding. Scheduling of messages according to user preference allowed for predictable receipt (eg, around mealtimes or avoidance of interruption at work), and repetition of information was linked to variety. Repetition of core messages in different ways was perceived as valuable but, crucially, only if the core message itself was valued. For many participants, the repetition of messages encouraging oral medication adherence was not perceived as valuable as so many participants had existing medication routines in place. In these cases, the repetition became problematic over time, and people talked about this with varying degrees of frustration and annoyance depending on whether they felt able to ignore these messages and remain engaged with the wider system.

## Discussion

### Principal Findings

This qualitative process evaluation identified a range of benefits of using a brief messaging system to support oral medication adherence for T2D. Overall, engagement with the intervention was high, it was used as intended, and people with T2D expressed positive views of the intervention, indicating that it was acceptable to participants as a supplementary digital health tool used over time. Users reported experiencing a range of holistic benefits, including direct changes to their behavior and changes in thoughts and feelings about their diabetes, and attributed this to receiving the messages over time.

Our study demonstrates the value of engaging people in thinking about their general health longitudinally. The analysis showed that the complex and fluid interplay among personal context, people’s existing self-management routines, and their beliefs about their condition appeared to influence appraisal of the system’s value. There was no clear evidence that contextual factors such as time since diagnosis, medication burden, or exposure to T2D education predicted whether people would perceive benefit from the system or, conversely, choose to stop receiving the messages. Echoing other work exploring the influence of digital tools on adherence [42], many people with established medication and lifestyle self-management routines reported feeling reassured; however, people with unstable routines described changes in both attitude and behavior whereby self-management and self-care became more consistent and automatic.

Value was attached to the regularity of message receipt over time, and particular benefit was reported by those participants willing to accept that not all messages would be of immediate relevance or usefulness. A basic threshold of relevance was not met for those who did not perceive value in the system. Patterns in descriptions of early impact appeared to remain relatively stable over time, indicating that initial benefit can be maintained and built upon through encouragement of sustained engagement with the system.

### Comparison With Existing Work

It is well documented that SMS text messages are considered to be an acceptable vehicle through which to communicate health information in the context of diabetes care. This study echoes previous work that has established that receipt of SMS text messages may mediate risk perception [43] and that messages with a positive tone and providing credible information with a degree of personalization can lead to improved self-monitoring and an increased perception of support [44-46]. This study contributes to the evidence on the delivery of contextually meaningful information to support T2D medication adherence and wider self-management.

In this study, provision of information and support that aligned with the users’ contextual needs and preferences was perceived as useful, relevant, and sustainable over time. It is broadly acknowledged that health information that is tailored to the individual is more effective than standardized or generic messages, with studies demonstrating modest positive improvement in glycemic control, particularly in poorly controlled diabetes [6,47,48]. Indeed, these effects may be sustained in the long term [49], with positive effects at least partially attributed to the provision of individually tailored support. However, while previous studies have demonstrated modest effects of tailored messaging grounded in specific user characteristics, they also acknowledge that population-level tailoring based on patient records is challenging and that exactly which criteria successful tailoring should be based upon remains unclear. Furthermore, interventions that are highly personalized and tailored to the individual result in highly complex interventions that are harder to implement consistently and whose efficacy is challenging to interpret [50]. Thus, *targeting* rather than tailoring of messages may be a more optimal use of resources. Targeted communication is intended to reach a population subgroup based on broadly shared and relevant characteristics, in contrast to tailoring, which is intended to reach specific individuals based on specific characteristics [51]. This study identified that people who perceived benefit from using the SuMMiT-D system did so by using the broadly targeted, moderately personalized information in ways that essentially allowed them to create their own bespoke messaging system that met contextually fluid needs and preferences. For some participants (eg, those with less stable existing routines), this might have resulted in adherence to treatment regimens for the first time, whereas for others, we propose that targeting supported a shift into what Schermer [52] terms “concordant self-management,” that is, the assimilation of one’s own knowledge of one’s condition with clinical recommendations to adopt an integrated self-management regimen. Furthermore, targeting based on self-determined interest, relevance, and perception of benefit (as described by SuMMiT-D trial participants) may contribute to increased engagement over time, particularly when combined with other modes of interaction such as web resources [53].

### Implications for Theory

SMS text messages may provide an effective delivery method for an intervention; however, to date, many existing SMS text messaging–based interventions do not specify a theoretical basis or propose specific mechanisms of action. Previous work in the SuMMiT-D project [29] has demonstrated how use of the SMS text messaging system can influence psychological constructs that predict adherence and that these were correlated with changes in self-reported medication adherence. The findings of our study further extend our logic model by proposing that mechanisms of action involved in habit formation are activated across the model. People described feeling supported in forming an intention to take medication, taking action necessary to take their medication, and monitoring this action to encourage consistency and recover from slips. Indeed, habit formation was described by participants in all areas of self-management, not just medication taking. Furthermore, while some of the changes described are in line with the mechanisms described in the SuMMiT-D logic model, additional characteristics of the system appear to have exerted an influence. These include the perceived “value” of the system to people regardless of how established their routines regarding medication were at the outset as well as a more generalized “activation” that people felt regarding their health and other behaviors not necessarily limited to diabetes and medication adherence.

It could be inferred that the content (eg, including evidence-based BCTs designed to support motivation and action control and increase confidence), its just-in-time relevance [54-56], and the way in which it was delivered (eg, regularly, in short messages, and over the course of a year) could have acted together in these cases to support participants’ self-regulation. Most participants reported consistent habits at baseline, meaning that these participants may not have needed support with intention formation or, potentially, habit formation. However, the interviews revealed more self-reported change than would be suggested by this and by our initial logic model. This indicates that low-burden, low-cost interventions such as a brief messaging system may benefit people *even if they do not perceive themselves as requiring support* by fundamentally supporting the cognitions, beliefs, and behaviors necessary for consistent self-management over time.

### Implications for Practice

For many complex health interventions, primary considerations of context focus on the health setting or person delivering the intervention [57,58]. In the case of the SuMMiT-D messaging system, a complex intervention was delivered quite literally into the back pocket of people’s lives. Thus, preexisting levels of knowledge, understanding, confidence, and motivation all affected the self-management actions that people were already taking either occasionally or habitually. In addition, participants’ fluid contexts influenced their subjective capacity to operationalize the resources at their disposal. This has important implications for implementation and the positioning of the SuMMiT-D system within the T2D self-management and information ecosystem.

SMS text messaging as a form of delivery could not and is not intended to replace more structured education courses for people with T2D. However, there could be potential for SMS text messages to support the retention of information from these courses (through repetition) and the long-term salience of this information as people deal with disruptions to routines, slipups, changing levels of motivation, and other potential barriers to continuing to effectively self-manage T2D in the long term. Supporting the cognitions, beliefs, and behaviors necessary for consistent self-management over time could be operationalized through the delivery of a wide variety of messages, and this variety of messages included in the library could perhaps increase the chance of the right message being delivered to a participant at the right time for them. It was indicated that those with established intentions and even established habits appreciated support and reinforcement of these by the SMS text messages over time.

These findings have implications for implementation—for example, messages would need to be introduced in the future so that people are encouraged to have realistic expectations about what they might “get” from such a system, what proportion of messages might be uniquely useful to them, and the fact that this might change over time. Instant messaging is likely to be used as part of or alongside disease management apps and wider programs, alleviating concerns regarding cost-effectiveness of delivery at scale [59]. These findings have helped us understand how the intervention was received by people over the course of a year and will be combined with quantitative process analysis of the main trial data to build a comprehensive program theory for this intervention that will help us understand how to optimize this intervention and how to implement it in the future so as to optimize the benefits identified in this analysis. Furthermore, this study also reiterates the need to reconceptualize the role of SMS text messages in the delivery of health care. It is common practice to use SMS text messages as a vehicle for reminders in primary care (eg, appointments and prescription refills), but this study (together with ample evidence articulating the ways in which SMS text messaging systems can be used to influence cognition and behavior) strongly indicates the value of reimagining our use of relatively simple, scalable, and accessible technologies [13].

### Strengths and Limitations

Process evaluations aim to understand how complex interventions bring about outcomes by exploring intervention mechanisms, context, and implementation. However, there are tensions between the perceived relative value of process evaluations in terms of the prioritization of a positive primary outcome versus understanding the richness of a participant’s context [30,60]. The updated Medical Research Council framework for evaluating complex interventions [20] states the following: “A trade-off exists between precise unbiased answers to narrow questions and more uncertain answers to broader, more complex questions; researchers should answer the questions that are most useful to decision makers rather than those that can be answered with greater certainty.” Furthermore, the “complexity turn” has highlighted the limitations of relying on causal inference alone for understanding whether and under which conditions interventions in complex systems “work” and what mechanisms might link interventions and outcomes [61,62]. Together, this implies that the pragmatic determination of the *holistic* value of an intervention will become increasingly significant. This is a key strength of the approach taken to the SuMMiT-D program of work, which fundamentally acknowledges the dynamic and interactive nature of context as explored through participants’ lived experiences [57,63]. Additional strengths of this study include its sample size, longitudinal design, and diverse population and that this was an objective a priori qualitative exploration of participant experiences that was data driven and not attached to explaining or justifying known quantitative outcomes.

The systematic approach that we used for data collection enabled us to build on what was known from formative work while exploring contexts and changes perceived as salient by the participants. In addition, we interviewed some participants at 2 time points, so we were able to gain an understanding of how early impressions of the system changed or stayed the same over time. Limitations to this study are acknowledged. Although some participants reported occasional lapses in medication-taking behaviors, few described having significant difficulties with oral medication adherence, and this may limit the broader application of the findings. Furthermore, there is inherent difficulty in asking participants to reflect on what would be termed *mechanisms of change* as it is generally difficult for a person to conceptualize why or how something might have changed in their self-management behaviors. Doing so assumes heightened self-awareness and requires detailed retrospective recall over the course of a year of participation in the trial.

### Conclusions

This process evaluation indicates that a targeted and automated SMS text messaging program has potential for increasing people’s self-management of their T2D. Such systems provide simple, accessible, scalable, holistic, and sustainable support. Participants in the SuMMiT-D study who perceived the system positively engaged with targeted content in a contextually fluid way based on relevance and need. They reported experiencing a range of holistic and unexpected benefits, including direct changes in their behavior and changes in thoughts and feelings about their diabetes, and attributed this to receiving the messages over time. This study suggests that a generic system with moderate targeting is acceptable and feasible and may offer benefits for a wide range of people, challenging assumptions about who might be expected to appraise such systems positively and who should be offered access to them.

## Data Availability

The datasets generated or analyzed during this study are not publicly available due to the qualitative nature of the data, which include sensitive and potentially identifiable information, but are available from the corresponding author on reasonable request.

## Abbreviations

BCT: behavior change technique
PPI: patient and public involvement
SuMMiT-D: Support Through Mobile Messaging and Digital Health Technology for Diabetes
T2D: type 2 diabetes
